# Anti-plasmodial action of *de-novo*-designed, cationic, lysine-branched, amphipathic, helical peptides

**DOI:** 10.1186/1475-2875-11-S1-P55

**Published:** 2012-10-15

**Authors:** Naveen K Kaushik, Jyotsna Sharma, Dinkar Sahal

**Affiliations:** 1 Malaria Research Group, International Centre for Genetic Engineering and Biotechnology, Aruna Asaf Ali Marg, New Delhi, 110067, India

## Background

A lack of vaccine and rampant drug resistance demands new anti-malarials under such circumstances antibiotic peptides may offer a novel approach to tackle the parasite.

## Methods

*In vitro* blood stage anti-plasmodial properties of several *de novo*-designed, chemically synthesized, cationic, amphipathic, helical, antibiotic peptides were examined against *Plasmodium falciparum* using SYBR Green assay. Mechanistic details of anti-plasmodial action were examined by optical/fluorescence microscopy and FACS analysis.

## Results

Unlike the monomeric decapeptides {(Ac-GXRKXHKXWA-NH_2_) (X= F,ΔF) (Fm ΔFm IC_50_ >100 µM)}, the lysine-branched,dimeric versions showed far greater potency {IC_50_ (µM) Fd 1.5 , ΔFd 1.39}. The more helical and proteolytically stable ΔFd was studied for mechanistic details. ΔFq, a K-K_2_ dendrimerof ΔFm and (ΔFm)_2_ a linear dimer of ΔFm showed IC_50_ (µM) of 0.25 and 2.4 respectively. The healthy/infected red cell selectivity indices were >35 (ΔFd), >20 (ΔFm)_2_ and 10 (ΔFq). FITC-ΔFd showed rapid and selective accumulation in parasitized red cells. Overlaying DAPI and FITC florescence suggested that ΔFd binds DNA. Trophozoites and schizonts incubated with ΔFd (2.5 µM) egressed anomalously and Band-3 immunostaining revealed them not to be associated with RBC membrane. Prematurely egressed merozoites from peptide treated cultures were found to be invasion incompetent.

## Conclusion

Good selectivity (>35), good resistance index (1.1) and low cytotoxicity indicate the promise of ΔFd against malaria.

